# Baseline Predictors of High Adherence to a Coitally Dependent Microbicide Gel Based on an Objective Marker of Use: Findings from the Carraguard Phase 3 Trial

**DOI:** 10.1007/s10461-015-1123-x

**Published:** 2015-07-24

**Authors:** Barbara A. Friedland, Marie Stoner, Michelle M. Chau, Marlena Gehret Plagianos, Sumen Govender, Neetha Morar, Lydia Altini, Stephanie Skoler-Karpoff, Khatija Ahmed, Gita Ramjee, Constance Monedi, Robin Maguire, Pekka Lähteenmäki

**Affiliations:** 1 Population Council, 1 Dag Hammarskjold Plaza, New York, NY 10017 USA; 2 Population Council, Johannesburg, South Africa; 3 South African Medical Research Council, Durban, South Africa; 4 University of Cape Town, Cape Town, South Africa; 5 Memorial Sloan Kettering Cancer Center, New York, NY USA; 6 Setshaba Research Centre, Soshanguve, South Africa; 7 Department of Obstetrics and Gynecology, University of Helsinki, Helsinki, Finland

**Keywords:** Microbicide, Carraguard, Adherence, Biomarker, Applicator test, Dye stain assay, South Africa

## Abstract

A randomized, placebo-controlled, efficacy trial of Carraguard was unable to demonstrate a reduction in women’s risk of HIV infection, which may have been due, in part, to low adherence (gel used in 42 % of vaginal sex acts, on average). A secondary analysis was undertaken to understand baseline factors associated with high adherence (gel used in ≥85 % of sex acts). Women who reported ≥1 vaginal sex act, returned ≥1 opened applicator, and had ≥1 conclusive post-enrollment HIV test (*N* = 5990) were included. Adherence was estimated as the ratio of average weekly applicator insertions (based on a dye stain assay indicating vaginal insertion)/average weekly sex acts (by self-report). Multivariate logistic regression modeling indicated that coital frequency, site, contraception, and partner age difference had a significant impact on adherence. Women reporting >1 and ≤2 vaginal sex acts per week, on average, were half as likely to be adherent as those reporting 1 vaginal sex act per week or less [adjusted odds ratio (AOR): 0.48; 95 % CI 0.38–0.61]; women from the Western Cape had one-third the odds of being adherent compared to women from KZN (AOR: 0.31; 95 % CI 0.23–0.41); compared to women using injectable contraception, women using any other or no method were more likely to be adherent (AOR: 1.30; 95 % CI 1.04–1.63); and women who had a larger age gap from their partners were more likely to be adherent (AOR: 1.03; 95 % CI 1.01–1.05; p = 0.001). Despite low adherence, overall, 13 % of participants achieved nearly perfect adherence, indicating a potential niche for a coitally dependent microbicide. More research is needed on the impact of sexual patterns and HIV risk perception on product acceptability and adherence to improve counseling in ongoing trials and when products are eventually introduced.

## Introduction

Globally, 60 % of new infections among 15–24 year olds occur in females, and 80 % of young women living with HIV and AIDS are in sub-Saharan Africa [1]. A host of biological and socio-cultural factors puts women, and young women, in particular, at greater risk of HIV acquisition than men [1, 2]. To arm women with an HIV prevention method within their control—or, at a minimum, that they can initiate—scientists have been developing vaginal microbicides [2]. Results of most trials conducted since the late 1990s have been disappointing. Large-scale trials of Savvy^®^ [3, 4], Carraguard [5], Pro-2000 gel [6, 7] and BufferGel [6] found no effect on HIV incidence. The Col-1492 trial of nonoxynol-9 demonstrated increased HIV risk and a Phase 3 cellulose sulfate (CS) trial was stopped early due to potential harm [8, 9]. To date, only CAPRISA 004, the first trial of an anti-retroviral (ARV)-based microbicide, tenofovir gel, has been able to demonstrate a significant (39 %) reduction in HIV risk in women [10].

More recently, failure to demonstrate effectiveness in large-scale trials of oral pre-exposure prophylaxis (PrEP) for women has been attributed to low levels of adherence. In the FEM-PrEP trial of once daily oral Truvada [tenofovir disoproxil fumarate (TDF) and emtricitabine (FTC)], drug levels indicated that less than 40 % of HIV-uninfected participants had evidence of recent pill use, leading to early closure of the trial [11]. Similarly, the Vaginal and Oral Interventions to Control the Epidemic (VOICE) study was unable to demonstrate effectiveness of two oral PrEP regimens (TDF-FTC or TDF alone) due to low adherence, estimated to be around 30 % [12].

Mathematical models have shown that a product’s effectiveness will be directly affected by the exact amount of product nonuse; for example, a 40 % *efficacious* product used in 50 % of sex acts would yield an *effectiveness* level of 20 % [13, 14]. The impact of adherence on effectiveness is illustrated by the dramatic difference in the outcomes of FEM-PrEP and VOICE compared to two other oral PrEP trials, Partners in Prevention [15] and the Botswana Oral PrEP study [16], both of which demonstrated a significant effect (62–75 %) in reducing the risk of HIV when adherence (based on drug levels) was estimated to be about 80 %, on average. The impact of adherence on effectiveness was also found in CAPRISA 004, in which tenofovir’s protective effect rose to 54 % among women using gel during more than 80 % of sex acts [10].

**Fig. 1:**
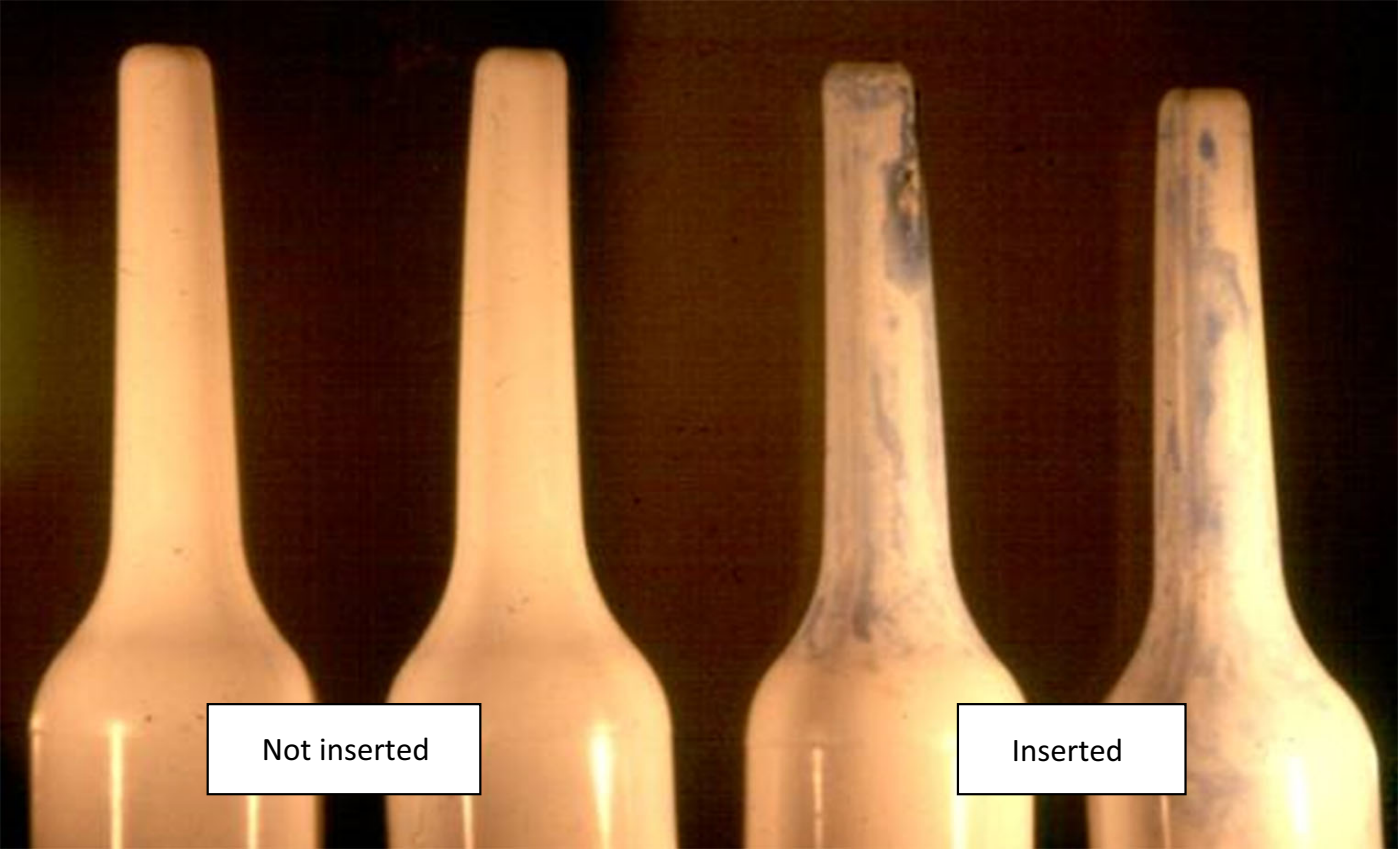
Dye stain assay for determining applicator insertion

Measuring adherence is complex, time-consuming, and requires considerable resources, even when biomarkers (such as drug levels in blood or urine) can be measured. Because none of the first-generation candidate microbicides was systemically absorbed, measuring adherence was even more challenging, as no biomarker could be used [17]. Therefore, participants’ self-reports were the primary adherence measure in numerous large-scale trials and were supplemented, in some cases, by counting [7] or weighing returned applicators [18–20]. The Carraguard^®^ Phase 3 efficacy trial was the first study to incorporate a dye stain assay (DSA) as an objective marker of applicator insertion. The DSA was developed and validated (97.5 % sensitivity, 96 % specificity) in preparation for the Phase 3 trial [21, 22]. In a review of several subsequent DSA validation experiments with other applicators and gels, Katzen et al. concluded that the DSA consistently yielded high (over 90 %) sensitivity and specificity on single-use applicators used once before sex [23]. The DSA has since been validated on Microlax applicators stored for up to four months [23] and used post-coitus [24]. The DSA involves spraying applicators with a solution made from blue food dye powder and water that reacts to vaginal mucous. Applicators exhibit a characteristic turquoise streaking pattern if they have been vaginally inserted (Fig. 1).

Based on the DSA results, gel was estimated to have been used in only 42 % of sex acts, overall, which may have contributed to the lack of protective effect found in the Carraguard Phase 3 trial [5]. In an effort to inform product development and to provide recommendations for future microbicide trials, an analysis was undertaken to identify baseline factors associated with high adherence in the Carraguard Phase 3 trial. Previous research on predictors of microbicide adherence has been limited, with varying results. In two small studies, one among women considered to be at high risk of HIV in the US (N = 96) who used a commercial lubricant for 2 weeks as a proxy for a microbicide [25], and another among female sex workers in Madagascar (N = 192) who used a diaphragm with or without a microbicide for 4 weeks [26], less “relationship power” (measured by partner resistance or violence related to suggested condom use) was associated with higher rates of adherence. By contrast, age (older women) and privacy (more than one room for sleeping) were associated with more consistent gel use among women enrolled with their HIV-positive partners (N = 544) at the Uganda site of MDP 301, a Phase 3 efficacy trial of Pro-2000 gel [27]. In a six-month study among monogamous Zimbabwean women (N = 117) using a microbicide gel with or without a diaphragm, positive partnership dynamics (partner approving of product use, consistent condom use) were predictors of consistent use [28], whereas no association was found between psychosocial variables (such as couple harmony, HIV risk perception) and consistent gel use among married women (N = 100) from the Pune, India, site of HPTN 059, a Phase 2 trial of tenofovir gel [29]. This is the first analysis to be undertaken using the DSA as an objective adherence marker (versus self-report) to evaluate baseline predictors of high adherence in a large-scale microbicide trial.

## Methods

### Carraguard Phase 3 Trial—Design and Population

The Carraguard trial was a Phase 3, randomized, placebo-controlled, double-blind trial to assess the efficacy of Carraguard, a carrageenan-based gel, in preventing HIV infection in women (N = 6202) conducted at three South African sites: Gugulethu [University of Cape Town (UCT)], Western Cape (N = 2315), Soshanguve (Medunsa), Gauteng (N = 2402), and Isipingo [Medical Research Council (MRC)], KwaZulu-Natal (N = 1485). Methods and results have been described in detail previously [5]. Briefly, participants were sexually active (defined as having had vaginal sex at least once in the 3 months before screening), HIV-negative, non-pregnant women 16 and older who were randomly assigned (1:1 ratio) to use Carraguard or a placebo (methylcellulose). Both gels were packaged identically in single-use Microlax-type applicators (Tectubes Sweden AB, Ǻstorp, Sweden), each filled with 5 mL to deliver a 4 mL dose. At enrollment, participants were given two boxes of gel applicators (15 per box) and were instructed to insert one dose of gel up to 1 h before each vaginal sex act, with no maximum limit; to insert a new applicator for each act, even if more than one act occurred within an hour; to insert a new dose if vaginal sex occurred more than 1 h after gel insertion; to use the gel only vaginally; and to use condoms with the gel.

Participants attended the clinic 1 month after enrollment and quarterly thereafter for a minimum of nine and a maximum of 24 months of follow-up. Each visit included a pelvic exam; testing for HIV, pregnancy, and sexually transmitted infections (STIs); counseling on HIV risk reduction and adherence; and an interviewer-administered, structured behavioral questionnaire (BQ). The BQ collected participants’ self-reported data on the number of vaginal sex acts in the 2 weeks before the visit, if study gel was used during the last vaginal sex act, if a condom was used during the last vaginal sex act, and whether unprotected oral or anal sex had occurred since the last study visit. The BQ also included questions about HIV risk factors (such as partner abuse, drug use, multiple partners, sex in exchange for money or goods) and intravaginal practices.

Participants were instructed not to wash their used applicators, to store each used applicator in an individual plastic bag (supplied by the study), and to return all used and unused applicators at each visit. Women were informed that applicators might be saved for future research, but were not told specifically about the DSA because of concerns that prior knowledge of applicator testing might affect women’s behavior, although the results would not affect ongoing participation. Gel resupply was determined individually during counseling sessions, based on previous gel use (number of opened applicators returned and participants’ self-reported use since the previous visit) and expected use in the next 3 months. All applicators that were returned opened (presumably used) were tested to confirm vaginal insertion using the DSA, which was introduced after training and validation at each site. Because of the time lag between study start (March 2004) and initiation of the DSA (November 2004), some applicators were stored for up to a year before being tested. Once the backlog of applicators had been tested, applicators were batch tested with the DSA on an ongoing basis throughout the remainder of the trial.

### Adherence Assessment

The primary adherence measure was “covered” sex acts or the percentage of vaginal sex acts in which gel was used. For each participant, the numerator, “average weekly insertions,” was derived by summing the total number of applicators inserted (per the DSA) and dividing by the number of weeks in the trial. The denominator, “average weekly vaginal sex acts,” was calculated by taking the average of the number of vaginal sex acts reported at each visit (per the BQ) and dividing by two (reports at each visit were for the previous 2 weeks). For example, a woman who participated for 52 weeks and inserted 50 applicators (per the DSA) would have an average weekly insertion of **0.96** applicators (50 inserted applicators overall/52 weeks in study). Assuming this participant reported 3, 1, 5, 0, and 4 vaginal sex acts (in the past two weeks) at her Months 1, 3, 6, 9, and 12 follow-up visits, respectively, her average weekly vaginal sex acts would be **1.3** ([3+1+5+0+4]/5 study visits/2 weeks). Therefore, the participant was adherent 74 % of the time (0.96 weekly insertions/1.3 vaginal sex acts, per week). For this analysis, participants were considered “adherent” if they used gel during 85 % or more of vaginal sex acts, corresponding to the adherence level required for 80 % power to detect a 33 % efficacious product [30], the aim of Carraguard Phase 3 trial.

### Statistical Analysis

This analysis included the 5990 participants (96.6 % of the enrolled population) who had at least one conclusive post-enrollment HIV test, returned at least one opened applicator, and reported at least one vaginal sex act, and excluded participants who had no DSA results recorded. Stata (version 12.1; StataCorp LP, College Station, TX) was used for all analyses. To examine differences in characteristics between adherent and nonadherent populations, Pearson chi-square (*x* ^2^) tests were performed for categorical variables and *t* tests were performed for continuous variables, using the significance level of p < 0.05. Continuous variables with a non-linear relationship to the dependent variable (covered sex acts) were converted to categorical variables [31]. Logistic regression was used to estimate the odds ratio (OR) and corresponding 95 % confidence intervals (CIs) for the relationship between predictors of interest and adherence. Reference categories were chosen to be those associated with higher adherence. Bivariate analysis was used to determine covariates for the final model based on inclusion criteria of p < 0.05. Manual, backward, stepwise elimination was used to develop the final model. Age group and condom use at last sex were retained in all models, regardless of significance, due to a priori assumptions that they were likely to be associated with adherence. Treatment group was included to account for study design. Income and education were not included in the final model, because data were only collected at exit from a random sample of participants (N = 1601) and inclusion of those variables would have reduced the analysis sample substantially.

### Ethical Approval

The protocol (Population Council No. 322) was reviewed and approved by the Population Council Institutional Review Board (NY, USA); the University of KwaZulu-Natal (KZN) Biomedical Research Ethics Committee for the Medical Research Council (MRC); the University of Limpopo, Research, Ethics and Publication Committee for Medunsa; the University of Cape Town (UCT) Research Ethics Committee; and the South African Medicines Control Council (reference no. 20031003); and is registered at ClinicalTrials.gov (NCT00213083). All participants gave written informed consent before screening and enrollment into the trial.

## Results

### Baseline Characteristics

As shown in Table 1, there were several significant differences in baseline characteristics between the adherent (N = 764) and nonadherent (N = 5226) participants. Most notably, the average weekly vaginal sex acts at baseline was 1.3 for adherent women versus 2.1 times per week for those who were nonadherent (p < 0.001). There were also significant differences between sites (p < 0.001); UCT had the lowest percentage of adherent women (4.7 %) and the MRC had the highest (20.3 %). Although there was no significant difference in average age, overall, between adherent and nonadherent women (30.9 and 30.3, respectively), there was a significant difference by age group; women 21–29 were the least adherent group compared to both younger (16–20 year olds) and older women (≥30 years old).

**Table 1:** Characteristics of Carraguard Phase 3 participants at screening by level of adherence (*N* = 5990)

Characteristic	Nonadherent (< 85 % sex acts with gel) *N* = 5226	Adherent (≥ 85 % sex acts with gel) *N* = 764	Total *N* = 5990	p value^a^
Demographics				
Average age (median, SD, range)	30.9 (29, 10.4, 16–72)	30.3 (28, 10.9, 16–66)	5990	0.117
Age group (years)				0.005
16–20	921 (84.0)	175 (16.0)	1096	
21–29	1771 (88.4)	233 (11.6)	2004	
30–38	1267 (87.5)	181 (12.5)	1448	
≥39	1267 (87.9)	175 (12.1)	1442	
Currently married/living as married	1649 (88.4)	216 (11.6)	1865	0.067
Average years of education^b^ (median, SD, range)	8.5 (9, 2.8, 0–12)	8.4 (9, 3.2, 0–12)	1593	0.828
Average monthly income, ZAR^b^ (median, SD, range)	1112 (800, 1059, 0–8000)	1044 (800, 860, 0–4500)	1594	0.418
Site				<0.001
UCT	2129 (95.3)	106 (4.7)	2235	
Medunsa	1942 (84.3)	363 (15.8)	2305	
MRC	1155 (79.7)	295 (20.3)	1450	
Reproductive health				
Average weekly vaginal sex acts (median, SD, range)	2.1 (1.5, 2.2, 0–21)	1.3 (1.0, 1.4, 0–13.5)	5990	<0.001
Ever pregnant	4290 (87.5)	614 (12.5)	4904	0.248
Contraceptive method (use of more than one method possible)				
Permanent method (sterilization or hysterectomy)	657 (88.0)	89 (11.9)	746	0.471
Injectable (DMPA or Net-EN)	2352 (90.4)	250 (9.6)	2602	<0.001
Oral contraception	403 (86.1)	65 (13.9)	468	<0.001
Male condom	886 (83.0)	181 (17.0)	1067	<0.001
Other^c^	97 (91.5)	9 (8.5)	106	0.184
None	1169 (83.4)	232 (16.6)	1401	<0.001
Regular menses	2990 (86.5)	464 (13.4)	3454/5989	0.015
Partnership characteristics				
Had more than 1 partner in past 3 months	440 (85.4)	75 (14.6)	515	0.198
Has a steady sexual partner	5167 (87.3)	750 (12.7)	5917	0.098
Age difference with steady partner (median, SD, range)	4.68 (4, 4.84, −22 to 39)	5.07 (4, 5.12, −20 to 36)	5915	0.041
Has other partner(s)	453 (83.3)	91 (16.7)	544	0.004
Steady partner has other partner(s)				0.001
Yes	893 (85.8)	152 (14.6)	1045/5917	
No	1761 (89.6)	204 (10.4)	1965/5917	
Don’t know	2513 (86.5)	394 (13.6)	2907/5917	
Abuse^d^ by any partner, ≤3 months	1812 (86.7)	277 (13.3)	2089	0.391
Ever forced sex	550 (86.5)	86 (13.5)	636	0.539
Other HIV risk factors				
Condom at last sex, any partner	2654 (88.0)	361 (12.0)	3015	0.068
Condom at last sex, steady partner	1772 (87.6)	250 (12.4)	2022/5916	0.518
Condom at last sex, other partner	284 (83.5)	56 (16.5)	340/544	0.034
Unprotected oral sex, ≤3 months	436 (88.1)	59 (11.9)	495	0.561
Unprotected anal sex, ≤3 months	105 (81.4)	24 (18.6)	129	0.044
Ever had sex for money	147 (89.6)	17 (10.4)	164	0.352
STI (non-HIV) detected at screening	1358 (87.9)	187 (12.1)	1545	0.373

Unless otherwise indicated, number (%) of participants is noted

*ZAR* South African Rand

^a^p values represent χ^2^ test for categorical variables and *t* test for continuous variables

^b^Data collected at exit from a random sub-sample of participants only (*N* = 1601)

^c^Includes female condom; IUD; diaphragm; traditional methods; other birth control. Types of contraceptives are recorded independently; therefore, counts may not add up to total N

^d^Includes physical, emotional, psychological and economic abuse

### Summary of Sexual Activity and Adherence During Follow-up

**Fig. 2:**
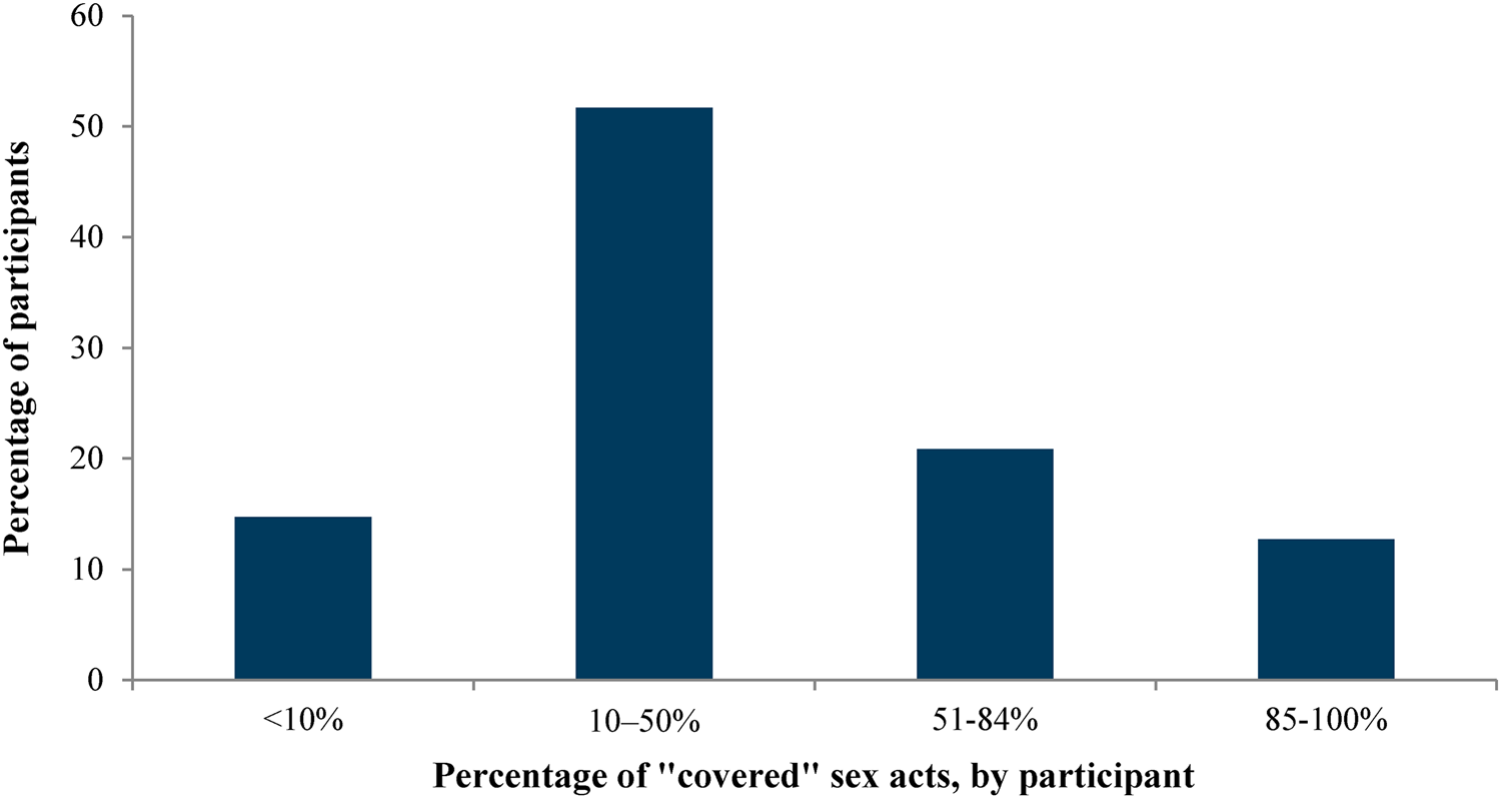
Distribution of average adherence (“covered sex acts”) by participant, Carraguard Phase 3 trial (*N* = 5990)

The mean length of participation, overall, was 1.31 years, with 30 % of women completing the maximum of 2 years. The average length of follow-up for adherent women was 1.08 years compared to 1.39 years for nonadherent women (Cochran-Mantel–Haenszel test of general association, p < 0.0001). Table 2 summarizes self-reported data on sexual activity, gel and condom use from the BQ and DSA results over the course of the trial. Overall, 90 % of applicators that had been issued were returned by participants, with no differences between those who were adherent or not. In addition, there was no difference by study group (Carraguard versus placebo) in the percentage of adherent versus nonadherent participants. According to DSA results, average weekly applicator insertions (total number of used applicators divided by the total number of weeks in the trial) ranged by participant from 0 to 9.5, with an average of 1.56 insertions among adherent women versus 0.79 insertions for nonadherent women (p < 0.001). The difference in average weekly vaginal sex acts between those who were adherent or nonadherent continued to be significant during follow-up; adherent women had a median of 1.2 acts per week compared to 2.4 acts per week among nonadherent women (p < 0.001). The percentage of “covered” sex acts (vaginal sex acts with gel) per participant ranged from 0 to 100 %, with an average of 42 %, overall. Adherent women used gel in 97 % of acts, on average (range 85–100 %), compared to only 34 %, on average (range 0–84.5 %) among nonadherent women (p < 0.001). Figure 2 further illustrates the distribution of “covered” sex acts, indicating that the majority of participants used the gel in no more than 50 % of their vaginal sex acts. Despite the range of adherence levels indicated by the DSA (Fig. 2), there were no differences between adherent (86.9 %) and nonadherent (85.3 %) women in the proportion reporting at all visits that they had used the gel during the last vaginal sex act.

**Table 2:** Summary of sexual activity, gel and condom use during the Carraguard Phase 3 trial, by adherence level (*N* = 5990)

Characteristic	Nonadherent (<85 % sex acts with gel) *N* = 5226	Adherent (≥85 % sex acts with gel) *N* = 764	Total *N* = 5990	p value^a^
Study group				0.983
Carraguard	2622 (87.3)	383 (12.7)	3005	
Placebo	2604 (87.2)	381 (12.8)	2985	
Average weekly coital frequency (median, SD, range)	2.7 (2.4, 1.6, 0–18.8)	1.3 (1.2, 0.6, 0–4.7)	2.5 (2.2, 1.6, 0–18.8)	<0.001
Percentage returned applicators (median, SD, range)	89.5 (97.5, 16.1, 1.7–100)	88.4 (97.8,18.5, 8.9–100)	89.4 (97.6, 16.4, 1.7–100)	0.085
Average weekly insertions per DSA (median, SD, range)	0.79 (0.71, 0.66, 0-4.1)	1.56 (1.45, 0.64, 0–9.5)	0.89 (0.79, 0.6, 0-9.5)	<0.001
Percentage vaginal sex acts with gel: inserted applicators [DSA]/sex acts [self-report] (median, SD, range)	33.8 (31, 21.8, 0–84.5)	97.3 (100, 4.7, 85–100)	41.9 (35.9, 29.4, 0–100)	<0.001
Self-reported gel at last sex				0.115
Always	4458 (87.0)	664 (13.0)	5122/5974	
Never	25 (78.1)	7 (21.9)	32/5974	
Inconsistent	728 (88.8)	92 (11.2)	820/5974	
Self-reported condom at last sex				0.004
Always	1599 (89.3)	192 (10.7)	1791	
Never	722 (85.0)	127 (15.0)	849	
Inconsistent	2905 (86.7)	445 (13.3)	3350	

Unless otherwise indicated, number (%) of participants is noted

^a^p values represent χ^2^ test for categorical variables and *t* test for continuous variables

In the adherent group, no difference in HIV incidence was found between those using Carraguard versus placebo; 20 in each group became infected with HIV (Risk Ratio ~ 1; p-value ~ 1.0). In addition, even among women estimated to have used the gel in 100 % of vaginal sex acts, 5 % of women in the Carraguard group (16 infections/331 women) and 5 % in the placebo group (15 infections/306 women) seroconverted (log-rank test, p = 0.80).

### Baseline Predictors of Adherence

In bivariate analysis (Table 3), age group, site, coital frequency (average weekly vaginal sex acts), site, contraceptive method, age difference with steady partner, having other partners, steady partner having multiple partners, and unprotected anal sex were associated with adherence. Women who had vaginal sex more than once but less than twice per week, on average, were less than half as likely to be adherent compared to women having vaginal sex once per week or less (OR: 0.46; 95 % CI 0.38–0.56), and the likelihood of being adherent declined as the average number of vaginal sex acts per week increased. Compared to participants from the MRC (KZN) site, participants from UCT (Western Cape) and Medunsa (Gauteng) had one-fifth and three-quarters the odds of being adherent, respectively. Participants who were not using any contraception at baseline were 1.5 times more likely to be adherent compared to those using contraception. Of those using contraception, women using methods other than injectables were more likely to be adherent than women using injectables (OR: 1.55; 95 % CI 1.29–1.86) and women who were not using condoms for family planning were less likely to be adherent than those using condoms (OR: 0.54; 95 % CI 0.45–0.66). Women were more likely to be adherent if they had multiple partners (OR: 1.42; 95 % CI 1.12–1.81) or if their partners had other partners (OR: 1.38; 95 % CI 1.17–1.64). Compared to women 39 years of age and older, women in the 16–20 year-old age group were significantly more likely to be adherent (OR: 1.38; 95 % CI 1.10–1.72), whereas there were no differences between the other age groups. Women who reported having had unprotected anal sex in the 3 months before screening were more likely to be adherent (OR: 1.58; 95 % CI 1.00–2.48), as were women with a greater age difference from their partners (OR: 1.02; 95 % CI 1.00–1.03).

**Table 3:** Bivariate logistic regression: baseline predictors of adherence (gel use in ≥ 85 % of sex acts) in the Carraguard Phase 3 trial (*N* = 5990)

Variable	Unadjusted odds ratio (95 % confidence interval)	Wald χ^2^ p value
Background characteristics		
Age group in years (vs. ≥39 years)		
16–20	1.38 (1.10–1.72)	0.006
21–29	0.92 (0.75–1.13)	0.648
30–38	1.03 (0.82–1.29)	0.766
Not married^a^ (vs. legally married/living as married)	1.17 (0.99–1.38)	0.068
Site (MRC reference)		
UCT	0.19 (0.15–0.25)	<0.001
Medunsa	0.73 (0.62–0.87)	<0.001
Carraguard (vs. placebo)	0.99 (0.86–1.16)	0.983
Reproductive health		
Coital frequency, last 2 weeks (vs. ≤1sex act per week)		
>1, ≤2 acts per week	0.46 (0.38–0.56)	<0.001
>2, ≤3 acts per week	0.21 (0.15–0.30)	<0.001
>3 acts per week	0.06 (0.03–0.10)	<0.001
Irregular menses (vs. regular)	0.86 (0.74–1.00)	0.066
Never pregnant (vs. ever)	1.12 (0.92–1.36)	0.248
Contraception^b^		
No sterilization/hysterectomy (vs. yes using)	1.09 (0.86–1.38)	0.471
No injectable (vs. yes using)	1.55 (1.29–1.86)	<0.001
No oral contraceptive (vs. yes using)	0.79 (0.60–1.05)	0.099
No condom (vs. yes using)	0.54 (0.45–0.66)	<0.001
Not using other (vs. yes using other forms)	1.59 (0.80–3.15)	0.188
None (vs. any contraception)	1.51 (1.28–1.79)	<0.001
Partnership characteristics		
No steady partner (vs. yes steady partner)	1.63 (0.91–2.94)	0.101
Steady partner age difference (years)	1.02 (1.00–1.03)	0.041
Steady partner has other partners (or doesn’t know) (vs. partner has no other partners)	1.38 (1.17–1.64)	<0.001
Participant has other partners (vs. no other partners)	1.42 (1.12–1.81)	0.004
HIV risk factors		
Did not use condom at last sex (vs. used)	0.90 (0.77–1.04)	0.155
Unprotected anal sex^c^ (vs. no unprotected anal sex)	1.58 (1.00–2.48)	0.046
Unprotected oral sex^c^ (vs. no unprotected oral sex)	0.92 (0.69–1.22)	0.561
Ever forced to have sex (vs. never)	1.08 (0.85–1.37)	0.540
Abuse^c^ (vs. no abuse)	1.07 (0.91–1.26)	0.391
Ever had sex in exchange for money (vs. never)	0.79 (0.47–1.31)	0.354
STI at screening (vs. no STI at screening)	0.92 (0.77–1.10)	0.373

^a^Not married = single/never married, divorced, separated, or widowed

^b^More than one method possible

^c^In the 3 months prior to screening

In the multivariate model (Table 4), coital frequency, site, injectable contraception, and age difference from steady partner remained significant predictors of adherence. Women having more than one and up to two vaginal sex acts per week, on average, had less than half the odds of being adherent as those having one or fewer vaginal sex acts per week [adjusted odds ratio (AOR): 0.48; 95 % CI 0.38–0.61]; those having more than two and up to three vaginal sex acts per week had approximately one-quarter the odds of being adherent (AOR: 0.24; 95 % CI 0.16–0.35); and those having more than three vaginal sex acts per week had less than one-tenth the odds of being adherent (AOR: 0.06; 95 % CI 0.03–0.11). Participants from UCT (Western Cape) remained significantly less likely to be adherent (AOR: 0.31; 95 % CI 0.23–0.41) compared to women from the MRC, although there was no longer a significant difference between women from the MRC and Medunsa sites (AOR: 0.98; 95 % CI 0.78–1.23). Women who had a greater age difference (in years) from their steady partners (AOR: 1.03; 95 % CI 1.01–1.05; p = 0.001) and those who were not using injectable contraception also had higher odds of being adherent (AOR: 1.30; 95 % CI 1.04–1.63). Age group was no longer a significant predictor of adherence.

**Table 4:** Multivariate logistic regression model: baseline predictors of adherence (gel use in ≥85 % of sex acts) in the Carraguard Phase 3 trial (*N* = 5990)

Variable	Adjusted odds ratio (95 % confidence interval)	Wald χ^2^ p value
Age group (vs. ≥39)		
16–20	1.16 (0.84–1.61)	0.368
21–29	0.88 (0.66–1.18)	0.406
30–38	1.08 (0.80–1.45)	0.616
Carraguard (vs. placebo)	0.94 (0.77–1.13)	0.49
Site (vs. MRC)		
UCT	0.31 (0.23–0.41)	<0.001
Medunsa	0.98 (0.78–1.23)	0.862
Average weekly vaginal sex acts (vs. ≤1 act per week)		
>1 and ≤2 acts per week	0.48 (0.38–0.61)	<0.001
>2 and ≤3 acts per week	0.24 (0.16–0.35)	<0.001
>3 acts per week	0.06 (0.03–0.11)	<0.001
Age difference with partner (years)	1.03 (1.01–1.05)	0.001
Not using injectable contraception (vs yes using)	1.30 (1.04–1.63)	0.021
Not using condoms for contraception (vs yes using)	0.84 (0.65–1.09)	0.188
Steady partner has other partners or does not know (vs partner does not have other partners)	1.10 (0.88–1.36)	0.404
Participant has other partners (vs no other partners)	1.20 (0.86–1.66)	0.280
Did not use a condom at last sex, any partner (vs. used a condom at last sex)	0.90 (0.73–1.12)	0.348
Unprotected anal sex, past 3 months (vs. no unprotected anal sex)	1.32 (0.73–2.39)	0.362

## Discussion

In this secondary analysis of data from the Carraguard Phase 3 trial, despite overall low rates of adherence (per the DSA), nearly 13 % of participants (764 women) achieved high adherence, having used gel in 85 % or more vaginal sex acts (N = 383, Carraguard; N = 381, placebo). However, even among this highly adherent group, there was no difference in HIV incidence between the Carraguard and placebo groups, consistent with the lack of protective effect found in the primary Carraguard Phase 3 trial analysis [5]. Regardless of Carraguard’s lack of effect, a better understanding of factors associated with high adherence in this trial may help to inform future clinical trials and the development of HIV prevention strategies for women, in general.

Coital frequency at baseline was one of the most significant factors associated with high adherence. The inverse relationship between coital frequency and adherence was also found by Turner et al., albeit among a cohort of female sex workers [26]. Participants (in both studies) were instructed to insert gel up to an hour before each vaginal sex act, and to insert a new dose for each act, even if multiple vaginal sex acts occurred within 1 hour. It is possible that women may not have been willing or felt the need to insert new applicators for each vaginal sex act. Results from a qualitative acceptability sub-study among Carraguard participants (N = 66) indicate the plausibility of this theory; of the women who participated in in-depth interviews, half of those reporting multiple “rounds” of vaginal sex inserted a new gel applicator for each vaginal sex act (N = 26/53) whereas the other half (N = 27/53) did not, either because they did not want to or because they thought the previously inserted gel would still be effective [32].

It is also possible that underlying patterns of sexual encounters rather than coital frequency, overall, led to the association between coital frequency and adherence. For example, if Participant X lives with her steady partner and regularly has vaginal sex once a week, she would report that she had two vaginal sex acts in the 2 weeks before her follow-up visit. If Participant Y, who does not live with her partner, spent the weekend before her study visit with him and had sex four times, she would have reported four vaginal sex acts in the previous 2 weeks. Therefore, even though both women had a total of four vaginal sex acts in the month preceding their study visits, based on the way data were collected (number of vaginal sex acts in the previous 2 weeks), coital frequency is estimated to be one vaginal sex act per week for Participant X versus two vaginal sex acts per week for Participant Y. Data from the qualitative Carraguard sub-study provide further evidence of the link between sexual patterns and adherence. In particular, many women reported that they forgot to bring their gels with them when they went to visit their boyfriends who were living elsewhere, while other women adjusted their behaviors to keep one box of gel at their boyfriend/partner’s house and another at home [32]. The impact of sexual patterns and logistics was also found to be associated with adherence issues in the recently completed FACTS 001 trial, in which most participants lived with their parents and sexual encounters often occurred outside of their homes [33].

The second factor that was consistently found to have a significant association with adherence was study site. Women from the UCT (Western Cape) site had one-third the odds of being adherent compared to those from Medunsa (Gauteng, near Pretoria) and the MRC (KZN), which may have been because they accurately perceived themselves to be at lower risk of HIV than women at the other two sites. Risk perception is a key element in behavioral change theories, such as the health belief model [34] and the AIDS risk reduction model [35]. Although HIV risk perception was not measured in the Carraguard Phase 3 trial, it is possible that women’s individual risk perception reflected country-wide surveillance data indicating that the Western Cape is the South African province with the lowest HIV prevalence [36]. Indeed, in comparison to the other sites, UCT had the lowest baseline prevalence [18 vs. 25 % in Medunsa (Gauteng) and 43 % at the MRC (KZN)] and incidence (2.7 infections per 100 woman-years versus 3.0 at Medunsa and 6.0 at the MRC) during the trial [5].

The significant difference in odds of adherence at UCT compared to the MRC and Medunsa sites may also have been due to logistical reasons. First, among a subset of women responding to quantitative, interviewer-administered exit interviews (N = 1601), significantly more women at UCT than the other two sites reported that the main reason for product nonuse was running out of study gel [37]. Second, mean length of participation was longer at UCT than at Medunsa or the MRC (1.35 years versus 1.29 and 1.26 years, respectively; Breslow-Day test, p = 0.03) [38] and adherence was negatively correlated with length of study participation. Shorter trials (1 year or less) and more frequent visits (monthly versus quarterly) may yield better adherence by minimizing study fatigue and the impact of resupply.

Contraceptive method was also found to be associated with adherence. Although injectable contraception was the most commonly used method, overall, women using any other method or no method at baseline had higher odds of adherence. Women using oral contraceptives (OCs) or condoms for family planning who were already accustomed to behaviors requiring daily or coitally related adherence may have been more comfortable incorporating a coitally dependent gel into their routines than women using injectables. These results are similar to findings from the Methods for Improving Reproductive Health (MIRA) trial, which evaluated the diaphragm for HIV prevention. In the MIRA trial, women reporting condom use at baseline were more likely to be adherent and women using injectable contraception, less likely [39]. On the other hand, in the FEM-PrEP trial of daily oral Truvada (or placebo), women using OCs at baseline were less likely to be good adherers, possibly because they may not have been willing to take a second pill every day [40].

In this analysis, partner age difference also had a significant association with adherence; the greater the age difference between a woman and her partner, the lower her odds of being adherent. Having an older partner (>5 years older) has previously been associated with increasing young women’s risk of HIV acquisition [36, 41], indicating that women at higher risk of HIV in the Carraguard trial, by virtue of having older partners, were less likely to be adherent than those at lower risk. These results align with van der Straten et al., who found that women in Zimbabwe at high HIV risk were less likely to be adherent to use of a diaphragm with a candidate microbicide [28], but differ from Mosack et al., who found that less “relationship power,” which was systematically assessed, was associated with greater adherence, albeit among a cohort of “high-risk” women in the United States [25]. Finally, in the FEM-PrEP trial, the first study of oral PrEP or vaginal microbicides to systematically assess the impact of risk perception on adherence, a significant positive association was found between having some perceived HIV risk at enrollment and good adherence [42].

The results of this analysis differ from the findings of Abaasa et al. [27], who found that older age and living in a household with more rooms for sleeping were associated with more consistent gel use at the Uganda MDP 301 trial site. The differences between the sites (rural Uganda in MDP 301 versus peri-urban South Africa in the Carraguard trial) and cohorts (discordant couples in Uganda versus sexually active women from the general population in South Africa), which tested similar broad-spectrum gels, both to be used within 1 hour of each sex act, highlights the importance of understanding the influence of contextual issues on adherence [17, 43].

### Limitations

There were several limitations to this analysis, and to the DSA, in particular. First, despite being a highly sensitive and specific measure of applicator insertion, the DSA cannot indicate if an applicator was inserted in conjunction with a specific vaginal sex act, nor whether gel was actually expelled into the vagina [44, 45]. Although training was conducted at each site prior to introducing the DSA, it is possible that readers became fatigued over time and, therefore, results could have been variable over the course of the trial. Additionally, because the assay was validated after the Phase 3 trial started, many applicators were stored for up to 1 year before being tested, which also could have affected the accuracy of the test. Finally, because applicator testing was not performed in connection to specific study visits, it was not possible to measure adherence by visit for each participant, but only to estimate adherence based average weekly insertions over the course of the entire trial. The lack of per-visit adherence measures also precluded an analysis of patterns of adherence over time.

Second, the measure of adherence is potentially flawed if self-reports of coital frequency were inaccurate. Women reported the number of sex acts in the 2 weeks prior to each quarterly visit, which was then extrapolated to the entire three-month period. If the denominator (number of sex acts) in the calculation of adherence was incorrect, it would have an impact on the overall ratio. Under reports of sexual activity would result in adherence being overestimated, while over reports of sexual activity would result in adherence being underestimated. If under and over reporting occurred with equal frequency, however, the outcome would be a higher estimate of adherence than actually occurred.

Results from a placebo gel trial at the same South African sites, however, in which self-reports and applicator testing both occurred monthly, indicated a similar percentage of sex acts with gel (44 % overall, over 3 months), with declining adherence over time [46]. Hence, it is conceivable that the overall level of adherence measured in this study (gel use in 42 % of sex acts) is accurate.

Finally, women who were not willing to abstain from intravaginal practices at screening were excluded from the trial. Once enrolled, women were asked quarterly about vaginal practices in conjunction with gel use (and were not discontinued from the trial); however, data on these practices were not collected at baseline. Therefore, it was not possible to assess the association between vaginal practices and adherence.

## Conclusion

The results of this analysis are important for the future of microbicide development. Given the overall low adherence to gel use in the Carraguard Phase 3 trial, alternative formulations, such as long-acting intravaginal rings or injectables may be more feasible for many women at high risk of HIV. The results of this analysis, however, in which over 700 women were able to achieve near perfect adherence, indicates that a coitally dependent gel is feasible for some women who may still be at high risk of HIV. The relationships between baseline coital frequency, contraceptive method and adherence in this large efficacy trial highlight the importance of integrating HIV prevention strategies into existing family planning programs and may inform the development of multi-purpose prevention technologies, designed to prevent pregnancy and HIV simultaneously. As illustrated by the between-site differences in this trial and results from other large-scale trials, no single product or formulation will work for all women. Therefore, it is important to continue developing multiple strategies that will be feasible for women at various stages of their lives.

This analysis can also help to inform future trials of microbicides and other HIV prevention strategies. Objective markers of adherence, such as the DSA, are extremely valuable for measuring adherence throughout a clinical trial, even when biomarkers are feasible. The ongoing use of biomarkers is prohibitively expensive; could potentially lead to a “white coat” effect, when participants use a product more regularly prior to a clinic visit; and cannot measure adherence in the control arm of placebo-controlled trials. However, even objective markers of adherence cannot fully explain the complexities of sexual patterns that may affect adherence. Future trials should consider including qualitative, in-depth assessments of women’s sexual patterns and HIV risk perceptions at baseline and throughout a trial to better predict potential adherence risks and to better counsel women who may have adherence challenges during a trial.

## References

[1] UNAIDS. The Gap Report. Geneva: Joint United Nations Programme on HIV/AIDS, 2014.

[2] Heise LL, Elias C (1995). Transforming AIDS prevention to meet women’s needs: a focus on developing countries. Soc Sci Med.

[3] Feldblum P, Adeiga A, Bakare R (2008). SAVVY vaginal gel (C31G) for prevention of HIV infection: a randomized controlled trial in Nigeria. PLoS One.

[4] Peterson L, Nanda K, Opoku BK (2007). SAVVY (C31G) gel for prevention of HIV infection in women: a Phase 3, double-blind, randomized, placebo-controlled trial in Ghana. PLoS One.

[5] Skoler-Karpoff S, Ramjee G, Ahmed K (2008). Efficacy of Carraguard for prevention of HIV infection in women in South Africa: a randomised, double-blind, placebo-controlled trial. Lancet.

[6] Abdool Karim SS, Richardson BA, Ramjee G (2011). Safety and effectiveness of BufferGel and 0.5% PRO2000 gel for the prevention of HIV infection in women. AIDS..

[7] McCormack S, Ramjee G, Kamali A (2010). PRO2000 vaginal gel for prevention of HIV-1 infection (Microbicides Development Programme 301): a phase 3, randomised, double-blind, parallel-group trial. Lancet.

[8] Van Damme L, Ramjee G, Alary M (2002). Effectiveness of COL-1492, a nonoxynol-9 vaginal gel, on HIV-1 transmission in female sex workers: a randomised controlled trial. Lancet.

[9] Van Damme L, Govinden R, Mirembe FM (2008). Lack of effectiveness of cellulose sulfate gel for the prevention of vaginal HIV transmission. NEJM.

[10] Abdool Karim Q, Abdool Karim SS, Frohlich JA (2010). Effectiveness and safety of tenofovir gel, an antiretroviral microbicide, for the prevention of HIV infection in women. Science..

[11] Van Damme L, Corneli A, Ahmed K (2012). Preexposure prophylaxis for HIV infection among African women. NEJM.

[12] Marrazzo J, Ramjee G, Richardson BA (2015). Tenofovir-based preexposure prophylaxis for HIV infection among African women. NEJM.

[13] Trussell J, Dominik R (2005). Will microbicide trials yield unbiased estimates of microbicide efficacy?. Contraception.

[14] Mâsse BR, Boily MC, Dimitrov D, Desai K (2009). Efficacy dilution in randomized placebo-controlled vaginal microbicide trials. Emerg Themes Epidemiol.

[15] Baeten JM, Donnell D, Ndase P (2012). Antiretroviral prophylaxis for HIV prevention in heterosexual men and women. NEJM.

[16] Thigpen MC, Kebaabetswe PM, Paxton LA (2012). Antiretroviral preexposure prophylaxis for heterosexual HIV transmission in Botswana. NEJM.

[17] Tolley EE, Friedland B, Gafos M, das Neves J, Sarmento B (2014). Socioeconomic and behavioral factors influencing choice, adherence and success of microbicide formulations. Drug Delivery and Development of Anti-HIV Microbicides.

[18] Kilmarx PH, van de Wijgert JH, Chaikummao S (2006). Safety and acceptability of the candidate microbicide Carraguard in Thai women: findings from a Phase II clinical trial. J Acquir Immune Defic Syndr.

[19] Kilmarx PH, Blanchard K, Chaikummao S (2008). A randomized, placebo-controlled trial to assess the safety and acceptability of use of Carraguard vaginal gel by heterosexual couples in Thailand. Sex Transm Dis.

[20] The Carraguard Phase II South Africa Study Team (2010). Expanded safety and acceptability of the candidate vaginal microbicide Carraguard^®^ in South Africa. Contraception.

[21] Wallace A, Thorn M, Maguire RA, Sudol KM, Phillips DM (2004). Assay for establishing whether microbicide applicators have been exposed to the vagina. Sex Transm Dis.

[22] Wallace AR, Teitelbaum A, Wan L (2007). Determining the feasibility of utilizing the microbicide applicator compliance assay for use in clinical trials. Contraception.

[23] Katzen LL, Fernández-Romero JA, Sarna A (2011). Validation of a dye stain assay for vaginally inserted hydroxyethylcellulose-filled microbicide applicators. Sex Transm Dis.

[24] Keller MJ, Buckley N, Katzen LL (2013). Use of the dye stain assay and ultraviolet light test for assessing vaginal insertion of placebo-filled applicators before and after sex. Sex Transm Dis.

[25] Mosack KE, Weeks MR, Novick Sylla MR, Abbott M (2005). High-risk women’s willingness to try a simulated vaginal microbicide: results from a pilot study. Women’s Health.

[26] Turner AN, Van Damme K, Jamieson DJ (2009). Predictors of adherent use of diaphragms and microbicide gel in a four-arm, randomized pilot study among female sex workers in Madagascar. Sex Transm Dis.

[27] Abaasa A, Crook A, Gafos M (2013). Long-term consistent use of a vaginal microbicide gel among HIV-1 sero-discordant couples in a phase III clinical trial (MDP 301) in rural south-west Uganda. Trials.

[28] van der Straten A, Moore J, Napierala S (2008). Consistent use of a combination product versus a single product in a safety trial of the diaphragm and microbicide in Harare, Zimbabwe. Contraception.

[29] Tolley EE, Tsui S, Mehendale S, Weaver MA, Kohli R (2012). Predicting product adherence in a topical microbicide safety trial in Pune, India. AIDS Behav.

[30] Lachin JM (1981). Introduction to sample size determination and power analysis for clinical trials. Control Clin Trials.

[31] Li AJ, Karlan BY. Breast cancer prevention. Obstet Gynecol. 2000;Special edition:18–22.

[32] Abbott S, Morar N, Madiba S, et al. Microbicides acceptability: the influence of social and cultural norms, interpersonal relations and sexual socialization. In: Presented at the Microbicides 2010 Conference, Pittsburgh, PA, May 22–25, 2010 [abstract 032].

[33] Rees H, Delany-Moretlwe S, Baron D, et al. FACTS 001 phase III trial of pericoital tenofovir 1% gel for HIV prevention in women. In: Presented at the Conference on Retroviruses and Opportunistic Infections (CROI), Seattle, Washington, February 23–26, 2015 [abstract 26LB].

[34] Rosenstoch I (1974). Historical origin of Health Belief model. Health Educ Monogr.

[35] Catania JA, Kegeles SM, Coates TJ (1990). Towards an understanding of risk behavior: an AIDS risk reduction model (ARRM). Health Educ Q.

[36] Shisana O, Rehle T, Simbayi LC (2009). South African National HIV Prevalence, Incidence, Behaviour and Communication Survey 2008: a turning tide among teenagers? Cape Town.

[37] Littlefield S, Gehret M, Friedland B, et al. An analysis of varying measures of adherence among women enrolled in the Carraguard^®^ phase 3 trial. In: Presented at the Microbicides 2008 Conference, New Delhi, India, February 24–27, 2008 [abstract 533].

[38] Agresti A (2010). Analysis of Ordinal Categorical Data.

[39] van der Straten A, Shiboski S, Montgomery ET (2009). Patterns and predictors of adherence to diaphragm use in a phase III trial in sub-Saharan Africa: a trajectory analysis. JAIDS.

[40] Corneli A, Deese J, Wang M (2014). FEM-PrEP: adherence patterns and factors associated with adherence to a daily oral study product for pre-exposure prophylaxis. JAIDS..

[41] Were E, Curran K, Delany-Moretlwe S (2011). A prospective study of frequency and correlates of intimate partner violence among African heterosexual HIV serodiscordant couples. AIDS.

[42] Corneli A, Wang M, Agot K (2014). Perception of HIV risk and adherence to a daily, investigational pill for HIV prevention in FEM-PrEP. JAIDS.

[43] Hayes R, Kapiga S, Padian N, McCormack S, Wasserheit J (2010). HIV prevention research: taking stock and the way forward. AIDS.

[44] Mauck CK, Schwartz JL (2012). Dyeing to know: the use of vaginal applicator staining and other techniques to assess adherence to product use in microbicide trials. Sex Transm Dis.

[45] van de Wijgert J, Jones H, Kilmarx PH (2009). Vaginal microbicide adherence biomarkers should be validated. Lancet.

[46] Mensch BS, Hewett PC, Abbott S (2011). Assessing the reporting of adherence and sexual activity in a simulated microbicide trial in South Africa: an interview mode experiment using a placebo gel. AIDS Behav.

